# The Impact of Different Sounds on Stress Level in the Context of EEG, Cardiac Measures and Subjective Stress Level: A Pilot Study

**DOI:** 10.3390/brainsci10100728

**Published:** 2020-10-13

**Authors:** Szczepan Paszkiel, Paweł Dobrakowski, Adam Łysiak

**Affiliations:** 1 Faculty of Electrical Engineering, Automatic Control and Informatics, Opole University of Technology, 45-758 Opole, Poland; ladam5@wp.pl; 2 Institute of Psychology, Humanitas University, 41-200 Sosnowiec, Poland; paweldobrakowski@interia.pl

**Keywords:** brain-computer interfaces, stress, measurement, sensors, neurofeedback, EEG, ASMR, Emotiv EPOC + NeuroHeadset, relaxation, stress relief

## Abstract

Everyone experiences stress at certain times in their lives. This feeling can motivate, however, if it persists for a prolonged period, it leads to negative changes in the human body. Stress is characterized, among other things, by increased blood pressure, increased pulse and decreased alpha-frequency brainwave activity. An overview of the literature indicates that music therapy can be an effective and inexpensive method of improving these factors. The objective of this study was to analyze the impact of various types of music on stress level in subjects. The conducted experiment involved nine females, aged 22. All participants were healthy and did not have any neurological or psychiatric disorders. The test included four types of audio stimuli: silence (control sample), rap, relaxing music and music triggering an autonomous sensory meridian response (ASMR) phenomenon. The impact of individual sound types was assessed using data obtained from four sources: a fourteen-channel electroencephalograph, a blood pressure monitor, a pulsometer and participant’s subjective stress perception. The conclusions from the conducted study indicate that rap music negatively affects the reduction of stress level compared to the control group (*p* < 0.05), whereas relaxing music and ASMR calms subjects much faster than silence (*p* < 0.05).

## 1. Introduction

Stress nowadays is very common, so much so that it has become one of the major problems of today’s society [1]. The phenomenon of chronic stress is affecting more people every year [2]. Brain–computer interface technology can be a helpful tool in the fight against stress. Hans Hugon Selye, who introduced the concept of stress, defines it as, “...a non-specific response of the body to a challenging situation.” Stress has been recognized as a state in which a person feels burdened with the requirements or a situation he/she finds themselves in [3]. One of the most common bases for this state, the so-called stressors, are moments which require acting under pressure and are observed primarily in interpersonal contacts, the workplace and situations associated with finances [1]. Therefore, stress is generated through animbalance between environmental requirements and an individual’s perception of their skills which are insufficient to satisfy those requirements [2,4].

Human biological functions are regulated by the two stress axes: the hypothalamus-pituitary-adrenal (HPA) axis and the sympathetic-adrenal-medullary (SAM) axis. The HPA axis is activated when the body perceives a physical or psychological stressor. Release of corticotropin (CRH) and arginine vasopressin (AVP) results in adrenocorticotropic hormone (ACTH) pituitary release. ACTH then stimulates the release of glucocorticoids from the adrenal cortex. SAM axis is a coordinated response to diverse stressors mediated by the release of epinephrine (from the adrenal medulla) and norepinephrine (from the peripheral sympathetic nerves). Crosstalk between the central nervous system and pituitary coordinate HPA and SAM axis activation and the pituitary β-endorphin release. Circulating glucocorticoids and catecholamines interact with a wide variety of cells to alter both metabolic and immune functions(Table 1).

The stress response leads to many physiological phenomena that can be measured. The reaction of the vascular system shows increased blood pressure. When a person is in a stressful situation, their heart rate and ECG wave pattern are affected. Stress can even trigger atrial and ventricular arrhythmias [5]. Hand sweat glands also increase their activity. The galvanic skin response (GSR) refers to changes in sweat gland activity. It changes the skin conductance, which is modulated autonomously by sympathetic activity and drives aspects of human behavior as well as cognitive and emotional states [6] and is not under conscious control. Skin conductance, therefore, offers direct insights into autonomous emotional regulation. During stress, skin conductance is higher [6]. Visualizing changes in brain bioelectrical activity requires more effort. However, it is important in order to register immediate changes, determine universality of the process (replicability) and for objectivity (difficulty in learning the reaction).

The goal of this research was to determine the impact of various types of sounds on lowering the stress level in a person, i.e., to confirm previous research and fill the knowledge gap of stress reduction potential of an autonomous sensory meridian response (ASMR) triggering sound. Specifically, this article attempted to answer the following questions: is it possible to satisfactorily test a person’s stress level? Do the sounds inducing ASMR (i.e., a phenomenon of relaxing formication) have higher relaxation potential than a musical genre so far considered as the most soothing (i.e., relaxing music)?

## 2. Stress under the Influence of Music

Short periods of stress can be considered motivating. The human body’s response to stress can vary, and symptoms can be considered dangerous if their duration is chronic. Persistent stress affects the body both physically and mentally [3,7]. One disease that stress contributes to is the cardiovascular disease, which aggravates by increasing blood pressure which is caused by a high level of adrenaline. A method to reduce stress level is something many people experience every day, not even aware of the fact that this can have such a significant impact on the stress reduction: listening to music [8]. Emotions induced by music engage brain structures which are responsible for regulating the hypothalamus, hypophysis and adrenal glands axis, which in turn is one of the most important stress systems within the human body [8]. Music is an essential part of human life and constitutes a good base for expressing and evoking specific emotions [3]. Individuals subconsciously select a particular music genre to change or maintain their emotional state or mood. Listening to music, as a method of reducing stress, is characterized as non-invasive, easy to implement and inexpensive, yet very efficient, owing to its ability to influence emotional, mental and behavioral changes [3,7]. This technique is effective, since brain structures responsible for perceiving emotions and music perceptions are located close to each other [3,5]. It is called music therapy and is applied as a technique supporting the treatment of various behavioral and mental diseases.

### Review of Previous Research

The correlations between reducing stress levels and music were the subject of numerous studies, described in a series of articles. The course of examination within the discussed field was described, among others, in [1]. The experiment was conducted on a group of women with musical education. Before the actual experiment, participants had to solve a mathematical problem from the field of arithmetic operations on four-digit numbers and were then asked to fill in a questionnaire on their current level of anxiety and arousal. Next, the subjects listened to various music genres and were again asked to complete a survey. The conducted experiments provided evidence that calm music reduced experienced stress to a greater extent than stimulating music. Moreover, the preferred music genre contributes to relaxing the subject more than the music he/she is familiar with but is not a fan of.

Another method of stress level reduction was applied by the authors of [8]. They looked at the results of cortisol level and alpha amylase from saliva samples collected from the study participants to identify stress. The experiment involved subjects listening to music for 30 min, five times a day, for seven consecutive days. The most interesting findings after the research work included the fact that listening to music significantly reduced stress if played for relaxation purposes. Otherwise the reduction probably occurred but was minimal. Another discovery was that listening in the company of others was more relaxing than listening alone. This technique was expanded in [4] by pulse rate measurement and a questionnaire. The first study stage was subjecting the participants to a stressful situation, which in this experiment was chosen as a job interview and solving arithmetic problems. Participants were divided into three different groups, two study groups and one control group. The control group was not subjected to acoustic stimulation with the sound of waves and relaxing music used in the experiments. The study provided evidence that the sound of waves relaxes subjects more than the relaxing music selected for the experiment.

Other researchers decided to check how familiar and preferred music listening affected the body [9]. They decided to measure stress using an ECG test and the subject’s breathing frequency. Each of the participants individually selected one track in the relaxing music and stimulating music genres. Next, the selected music was played to the subjects and measurements were taken. The experiment showed that relaxing music induced relaxing and stimulating emotions, whereas stimulating music increased the level of joyful emotions but did not impact the level of relaxation among participants.

The authors of [10] conducted their study using a musical genre which had not been previously selected for experiments in this field. They chose a representative of the Eastern culture (i.e., pieces played on the traditional Chinese instrument called the guqin) which is used during meditation. Participants were people without musical education. The study involved comparing the alpha-wave frequencies in the subjects listening for guqin, murmurs and silence. The experiment showed that Eastern music noticeably impacted the alpha-wave frequency synchronization compared to the other groups.

Other researchers described the application of EEG as a method of identifying neural correlates of emotional responses to music [11]. The experiment involved people of varied musical experience. The study consisted of six series of EEG records and a questionnaire on the emotional scope of the subjects. It showed that the right hemisphere was more active when experiencing negative feelings, whereas the left one was more active when experiencing positive ones.

The impact of Hindu music on brain waves was described in [12]. Ten men took part in the experiment and were subjected to testing in three different conditions, i.e., listening to chayanat music (joyful), darbari music (sad) and without sound stimulation. The study concentrated on the frontal lobes of the participants and analyzed the alpha, theta and gamma waves. It was observed that the alpha-wave frequency in the frontal lobes was low in right sided electrodes when listening to joyful music and in left sided electrodes while listening to sad music. No significant connection between the theta and gamma waves and the experienced emotions was observed.

Another research paper which combines the issues regarding the correlation between stress and music, is [13]. Its objective was to compare the impact of the participants’ favorite music with pre-selected relaxing sound material containing synchronous rumbling. The EEG examination was conducted using a 14-channel electroencephalograph. It was observed that the relaxing music intensified the alpha-wave level more than the participant’s favorite genre. That means that the relaxing music was more soothing for the respondents. Blood pressure and pulse also decreased more when listening to a track with synchronous rumbling, which confirms its relaxing properties.

The authors of [3] decided to study the difference between English language music and music in their native language (Urdu). The experiment was conducted using a 4-channel EEG device by MUSE, which automatically neutralized interference. The test results indicated that the genre of the music one listens to did not significantly contribute to a change in stress level, whereas the language of the song did. Furthermore, it was shown that women were more susceptible to emotional change when listening to music compared to men.

These studies showed how music is correlated with emotions experienced by an individual. One such feelings is stress which, with its correlation to music, constitutes the topic of this article. Coherent conclusions in terms of how the human body reacts to stress were drawn based on numerous experiments [13]. It has been proven that a relaxed human has a lower pulse and blood pressure compared to someone under stress. Furthermore, the experiment also indicated that systolic blood pressure more accurately reacts to increased/decreased stress levels than diastolic blood pressure and heart rate [13].

Music, as a therapeutic tool, was described in [14]. Authors used rhythmic music to augment treadmill training in Parkinson’s disease patients. Positive physical effects of training were amplified. However, influence on patient’s cognition was not clear.

In [15], authors studied response to music stimuli independently of patient’s consciousness using fMRI. To formulate gradient of consciousness, tests were done on nine post-comatose patients and eight healthy ones. Results showed that music stimulation increased connectivity in regions involved in consciousness, language, emotion and memory.

Work on cardiac autonomic response is also the subject of much research. Research currently underway evaluated the effects of acute battling rope exercise on heart rate variability and blood pressure (BP) responses in young men with elevated BP [16]. Heart rate variability (HRV) analyses can be performed using group or individual changes. Individual changes could be of potential interest during training camps for national soccer teams. The goal of one group of researchers was to compare whether analysis of individual daily HRV could detect changes in cardiac autonomic responses during training camps for national soccer teams. During two different training camps, 34 professional soccer players were monitored daily over nine days using heart rate monitors [17]. The autonomic nervous system (ANS) plays a key role in maintaining physiological homeostasis, and research with neurotypical and autistic individuals has found relations between cardiac autonomic responses as well as awareness of one’s cardiac responses and social and emotional processing. The aim of another group of researchers was to examine relations between cardiac autonomic activity, heartbeat perception, emotion processing and levels of autistic traits in a group of college students. Cardiac ANS at baseline and during an emotional picture task was measured, and a heartbeat perception task was used to assess interoceptive accuracy (IA) [18].

As it results from the analysis of the cited literature, some researchers attempted to study the impact of emotions on the human brain. The conclusions drawn includes the fact that cortical and subcortical networks of the central nervous system participate in controlling emotional states. Another indication was the dominance of one of the hemispheres when experiencing various states, with the right one leading in the case of negative states and the left one leading for positive states [3,19,20]. The researchers decided to analyze the bands of brain wave intensifying upon specific feelings experienced by the participants [21]. Electroencephalography recordings have been assessed as objective markers of consciousness with the presence of alpha and beta waves. Delta and theta waves are characteristic to unconsciousness [3]. Undoubtedly, the waves considered as stress level identifiers were alpha and beta [3]. When it comes to waves in the 12–32 Hz range, those frequencies were considered a characteristic indicator of decreasing relaxation level [3]. Significantly more tests have been completed for waves in the 8–12 Hz frequency range. The most common conclusion was that increased alpha-wave activity in frontal lobes was associated with the relaxation state and increased cognitive functions. Furthermore, those waves were considered as correlated with the music processing. The scientists selected listening to music as an activity, which decreased experienced stress, because of the surprisingly good effects compared to costs [4]. The experiments showed that listening to music contributed to a decrease in the stress perceived by a person and depending on the genre, it could lower his/her blood pressure and pulse and increase alpha-wave activity [3,9]. Coherent conclusions in terms of how the human body reacts to stress were drawn based on numerous experiments. It has been proven that a relaxed human has a lower pulse and blood pressure compared to someone under stress.

## 3. Materials and Methods

### 3.1. Practical Experiment and Participants

The study involved nine females. This choice was dictated by a statistically higher reactivity of women to the emotional state induced by music, as demonstrated in 2019 using EEG signals for human stress classification in response to music tracks [1]. All participants were right-handed and 22 years of age. All participants were healthy and did not have any neurological or psychiatric disorders. Before the study, the inclusion criteria for the study group was applied. There are many factors that ultimately affect the stress response and response to anti-stress protocols. Therefore, initial stress level, information on their menstrual cycle, BMI value, declarations not to take medications, not smoking and not consuming alcohol and caffeine on the day preceding the study were taken into account. The experiment was conducted on participants without musical education.

The duration of this experiment was 4 days, with the type of sound stimulation replaced each successive day by another musical genre, with the rest of the testing remaining the same. The music genres used in this experiment were no musical stimulation (silence, treated as a control group), relaxing music (made up of forest ambience), rap and ASMR-triggering music. At specific stages, the participants were subjected to a complete set of tests (blood pressure and pulse measurement) and were asked to fill in a questionnaire. Throughout the entire experiment, the EEG results of the subject were monitored, and particular attention was paid to the presence of alpha-waves in the signal. During the EEG examination, people were blindfolded and asked to remain still for the entire duration. This was aimed at minimizing the data reading interference resulting from movements. The survey received by the subject consisted of the question, “how stressed do you feel right now (on a scale from 1 to 10)?”

There were three points during the day in which measurements were taken. The first one was before any stressor or musical stimulation (this point is called *A* in the rest of the paper). Then, the participants were put in a stressful situation (i.e., performing complicated mathematical calculations). The participants were asked to present the highest possible number of correct solutions within a set time. In the event of a participant giving an incorrect result, she received this information straight away in order to increase the stress level even more. After this test, the blood pressure and pulse were measured again, and she was asked to complete a survey (point *B*). The next step was exposing the subject to a 5 min sound stimulation, followed by repeated measurements and questionnaire (point *C*). These tests were completed each day.

Table 2 shows the flowchart of the experiment. The suggested tests consist of repeating elements, in order to conduct an accurate analysis of the collected data.

### 3.2. Measurement Devices

A 14-channel Emotiv EPOC+ Neuroheadset electroencephalograph, blood pressure monitor, pulsometer and a questionnaire were used in the course of the research work [22,23]. The device for measuring blood pressure and heart rate was Tech-Med TMA-500PRO with a pressure measurement range of 30–280 mmHg and a pulse measurement range of 40–199 beats/minute. The task of each component was to determine the stress level experienced by a subject at a given moment.

Emotiv EPOC+ Neuroheadset is a wireless, non-invasive measuring device, used to monitor brainwave activity in the bandwidth of 0.16–43 Hz and digital notch filters at 50 Hz. Frequency measures were obtained using FFT with a 256 sample (2 s) Hanning window. The EEG signals were sampled with 128 Hz. The electrodes in this electroencephalogram are fixed in the following positions: AF3, AF4, F3, F4, F7, F8, FC5, FC6, T7, T8, P7, P8, O1, O2, while the reference electrodes are located at: P3 and P4 [23]. Alpha waves were identified at the O2 electrode, as they seem to have the biggest amplitude in the occipital lobe [24].

The individual frequency ranges were determined based on the subject’s peak alpha frequency (PAF) [25]. Frequency showing the highest spectral amplitude within 7 to 15 Hz band was identified as the PAF. Lower and upper limits of the alpha band were determined where “eyes open curve” crossed the “eyes closed curve” closest to the PAF (increases over right centrotemporal (C4, T4), left frontal (F3), and occipital (O1)) [26].

Blood pressure, pulse rate and subjective stress level were logged after every phase (A, B and C) [27]. To obtained subjective stress level in the fastest and most authentic way possible, the questionnaire contained only one question, which was “how stressed do you feel right now (on a scale from 1 to 10)?”

### 3.3. Numerical Measures: CBA Ratio and the Statistical Analysis

To determine the impact of music genre on the stress level, ratio $\frac{C-B}{A}$ was obtained for every measurement. It shows how big the difference between the music listening phase (*C*) and the stressor phase (*B*) is compared to the reference value (*A*), so the value before the measurement. In other words, it measures how fast one can relax given the “normal” state. In the rest of the paper, this measure will be called CBA ratio.

To test the hypothesis that the stress response is different for tested music genres, the Kruskal–Wallis test (ANOVA on ranks) was conducted, followed by Dunn’s multiple comparison test differentiating every music genre pair. Those tests were used, since the chi-squared test indicated no normality of obtained data. 

## 4. Results

**Figure 1:**
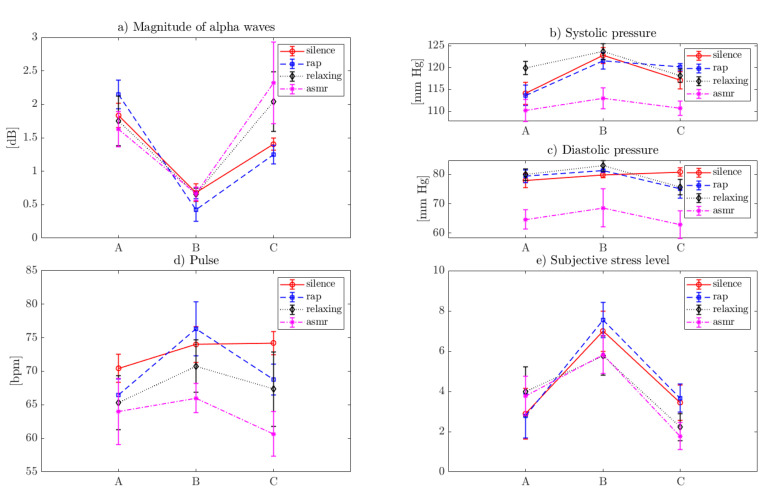
Visualization of the measurements (i.e., the mean value and the standard deviation). (**a**) The EEG measures; (**b**) and (**c**) are the pressure measure; (**d**) pulse; and (**e**) subjective stress level.

The mean values and the standard deviation of data collected during the experiment are shown in Table 3 and Figure 1.

**Figure 2:**
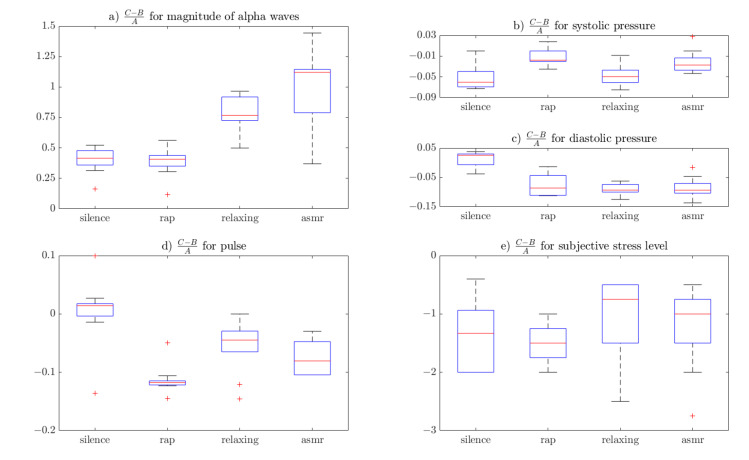
Visualization of the CBA ratio: (**a**) EEG measures; (**b**) and (**c**) pressure measure; (**d**) pulse; and (**e**) subjective stress level.

The results of the CBA ratio are shown in Table 4 and in the form of a boxplot in Figure 2. In the boxplot, the red line indicates the median, the blue box indicates the interquartile range and the whiskers represent the remaining values other than outliers which are indicated by red crosses.

Results of statistical tests, with corresponding statistical significance levels (*p*-values) are shown in Table 5.

## 5. Discussion

Measures taken for the silence period, or the reference group, indicate that stress vanishes spontaneously after some time. This is to be expected, as it is easy to experience in everyday life. Obtained CBA ratios, however, suggest that different music stimulations could hypothetically speed up or slow down the relaxation process.

It could be raised noted that without specific analysis of relaxation processes, conclusions about its dynamics cannot be soundly established. However, the CBA ratio was designed specifically regarding dynamics, by using the difference between the first (*B*) and the last (*C*) moment of relaxation, so just before and after music stimulation. Research on the dynamics of the relaxation process could be the subject of another research project.

It can be seen that pressure and pulse were affected by the experiment much less than the alpha waves and the subjective stress level. Also, for some music genres (i.e., relaxing and ASMR), the *C* levels indicated less stress than the reference *A*. That means that those genres were able not only to neutralize the stress effect, but to relax the subject to a calmer state than before the stressor.

The CBA ratio for magnitude of the alpha waves suggests that the biggest influence on the subject’s ability to relax can be attributed to ASMR sounds and relaxing music. It also shows that ASMR-triggering sounds are capable of making the participants even more relaxed than their pre-stressor state (CBA ratio greater than 1). Relaxing music looks somewhat worse, however statistical analysis shows no significant difference between those two (*p* > 0.05). Both ASMR-triggering and relaxing music shows significant difference compared to silence (*p* < 0.05) for the magnitude of the alpha waves. Rap music, on the other hand, shows no significant difference compared to silence (*p* > 0.05).

Pulse measurements points to opposite conclusions, with rap showing the biggest difference compared to the reference group. ASMR also seems somehow “worse” than the reference (*p* < 0.05). Relaxing sounds are not statistically different from silence. This suggests that relaxing sounds or no stimulation at all can be the best way to stabilize the pulse after a stressful situation.

Systolic pressure also suggests rap as the least efficient way to calm down. Surprisingly, the boxplots for CBA ratio of systolic pressure for rap and ASMR are very similar and visually different from the reference group. Relaxing music seems closest to the silence, however there is a statistical difference between them (*p* < 0.05).

Visual analysis (i.e., the boxplots and statistical tests of diastolic pressure) show a difference only between rap-reference and rap-relaxing pairs (*p* < 0.05 for both). Surprisingly, in terms of subjective stress level, all groups seem similar. Statistically, there is no difference between them in terms of the CBA ratio (i.e., music stimulation does not change subjective stress level expressed in CBA ratio form). 

The CBA ratio is a measure that shows how fast one can relax in any given circumstance. It can be raised that the denominator could also be normalized by the *B* value, creating a $\frac{C-B}{B-A}$ ratio. However, it would be uninterpretable when the stressor phase would not stress the participant at all, leaving zero in the denominator. Then again, putting the nominator and the denominator in (for example, logarithm) would remove this issue. The ratio, however, would be much less interpretable. On the other hand, maybe this kind of measurement should be excluded from the dataset since the participants would not be stressed enough to give interpretable results. Perhaps different stressors should be applied to them. This issue should be taken into consideration in future studies.

In the future, it will also be possible to assess the response of the autonomic nervous system. In this regard, bio-signals such as EKG (electrocardiography) and GSR (galvanic skin response), which can be easily and discreetly distinguished may prove useful. It is also planned to use more structured questionnaires for this type of study. For example, STAI-Y1 and POMSmay be useful in this regard.

In addition, it is worth noting an interesting issue in the future would be to conduct an experiment in which participants would freely choose the most relaxing music piece for them. Emotions induced by music are generally very subjective, as they are dependent on a lot of different associations. However, ASMR-triggering sound can hardly be considered music and is less susceptible for subjective connotations.

## 6. Conclusions

In this study, nine participants were examined and the effect of music stimulation on stress reduction was investigated. In an experiment of this type, the decrease in brain alpha-wave frequencies and a simultaneous increase in blood pressure and pulse of the participant can be observed in a stressful situation. Analogously, such tendencies, though opposite (i.e., increasing activity of a given wave and decreasing blood pressure monitor results) should be experienced in a state of relaxation.

An interesting conclusion that comes to mind after the completed tests is the fact that ASMR-triggering music reduces the stress level among people equally well as relaxing sounds. It was also observed that stress reduction is highly impacted by passing time. Such a conclusion can be drawn after analyzing and comparing the results obtained in the case of no stimulation and stimulation with rap music. The analysis indicates that this type of sound has the least effect on stress reduction in the subjects and even delays their relaxation. One of the observations is the correct selection of the stressor imposed on the test participants.

It could be beneficial to have some relaxing music or ASMR-triggering sounds saved on smartphones which could be listened to for a few minutes in a stressful situation. 

## Figures and Tables

**Table 1:** Scheme of interactions changing metabolic and immunological functions.

**Hypothalamus**	→ ←	**Central Nervous System**	
Pituitary		Adrenal Medulla	Peripheral Sympathetic Nerves
Adrenal Cortex		Epinephrine	Norepinephrine
Glucocorticoid		Metabolic Glycolysis	
Metabolic Glycolysis/gluconeogenesis Anabolism/catabolism Lipolysis Insulin signaling#		Immune Regulation of cytokines Stabilization of cytoskeleton Infection Wound healing	
Immune Apoptosis Neutrophil/lymphocyte trafficking Cytokine production Anti-inflammatory responses Pro- inflammatory responses		Behavior Aggressive behaviors Increased heart rate and blood pressure Increased respiration	

**Table 2:** Stages of the experiment.

Stage	Activity
1.	Filling in the questionnaire, measuring pulse and blood pressure, EEG examination (called *A* stage for short in the rest of the paper).
2.	Monitoring values achieved by alpha waves until the end of the test.
3.	Putting a subject in a stressful situation.
4.	Filling in the questionnaire, measuring pulse and blood pressure (called *B* stage for short in the rest of the paper).
5.	Playing a selected sound to the subject.
6.	Filling in the questionnaire, measuring pulse and blood pressure (called *C* stage for short in the rest of the paper).
7.	The end of the test.

**Table 3:** The mean and standard deviation of measured values.

Sound Type	A (Prior to Stressor)		B (After the Stressor)		C (After the Sound)	
	Mean	std	Mean	std	Mean	std
Mean alpha wave amplitude value [dB]						
Silence	1.83	0.18	0.73	0.14	1.40	0.09
Rap	2.12	0.20	0.47	0.21	1.25	0.14
Relaxing	1.48	0.42	0.66	0.09	1.67	0.60
ASMR	1.46	0.33	0.66	0.10	1.92	0.84
Systolic pressure [mm Hg]						
Silence	115.00	2.06	122.78	1.86	117.89	1.27
Rap	113.89	2.20	121.78	1.48	120.00	0.87
Relaxing	119.89	1.54	123.22	1.72	118.22	1.30
ASMR	111.78	1.79	113.67	2.18	111.00	1.50
Diastolic pressure [mm Hg]						
Silence	77.89	2.52	80.00	1.00	80.33	1.50
Rap	79.33	2.18	81.22	0.44	75.11	3.14
Relaxing	79.89	1.96	82.89	1.83	76.11	2.20
ASMR	69.11	5.60	76.00	8.62	67.33	6.12
Pulse [bpm]						
Silence	70.44	2.07	74.00	2.65	73.11	2.26
Rap	67.89	2.89	76.22	3.96	69.67	2.92
Relaxing	65.89	4.26	71.00	3.97	67.33	5.52
ASMR	64.22	5.09	66.56	2.51	61.33	3.71
Subjective stress level [1–10]						
Silence	2.78	1.20	6.89	0.93	3.11	0.93
Rap	2.67	1.00	7.72	0.67	4.11	0.78
Relaxing	3.83	1.17	6.00	0.83	2.22	0.67
ASMR	3.61	0.86	6.06	0.77	1.78	0.67

**Table 4:** The mean and the standard deviation of the CBA ratio.

Sound Type	Mean Alpha Wave Amplitude Value [dB]		Systolic Pressure [mm Hg]		Diastolic Pressure [mm Hg]		Pulse [bpm]		Subjective stress level [1–10]	
	Mean	std	Mean	std	Mean	std	Mean	std	Mean	std
Silence	0.394	0.107	−0.050	0.024	0.013	0.028	0.002	0.061	−1.554	1.062
Rap	0.382	0.122	−0.013	0.016	−0.077	0.039	−0.113	0.026	−1.491	0.347
Relaxing	0.781	0.152	−0.046	0.021	−0.090	0.019	−0.055	0.047	−1.054	0.737
ASMR	0.985	0.314	−0.021	0.022	−0.087	0.037	−0.086	0.054	−1.220	0.723

**Table 5:** The *p*-values of statistical hypotheses tests. The null hypothesis was that there is no significant difference between compared groups.

Statistical Difference	*p*-Value				
	EEG	Systolic Pressure	Diastolic Pressure	Pulse	Subjective Stress
Kruskal–Wallis	6.0 × 10^−5^	1.9 × 10^−3^	4.5 × 10^−4^	6.4 × 10^−4^	2.3 × 10^−1^
Silence—rap	1.0 × 10^0^	7.0 × 10^−3^	5.5 × 10^−3^	3.5 × 10^−4^	9.9 × 10^−1^
Silence—relaxing	2.8 × 10^−2^	2.3 × 10^−3^	1.0 × 10^0^	3.7 × 10^−1^	6.9 × 10^−1^
Silence—ASMR	2.5 × 10^−3^	2.3 × 10^−3^	1.4 × 10^−1^	3.6 × 10^−2^	9.8 × 10^−1^
Rap—relaxing	1.3 × 10^−2^	1.0 × 10^0^	1.8 × 10^−2^	1.5 × 10^−1^	2.7 × 10^−1^
Rap—ASMR	1.1 × 10^−3^	1.0 × 10^0^	8.7 × 10^−1^	7.4 × 10^−1^	7.2 × 10^−1^
Relaxing—ASMR	9.8 × 10^−1^	1.0 × 10^0^	3.0 × 10^−1^	9.2 × 10^−1^	9.9 × 10^−1^
